# Inside out *Porphyridium cruentum*: Beyond the Conventional Biorefinery Concept

**DOI:** 10.1021/acssuschemeng.2c05869

**Published:** 2022-11-29

**Authors:** Davide Liberti, Paola Imbimbo, Enrica Giustino, Luigi D’Elia, Giarita Ferraro, Angela Casillo, Anna Illiano, Gabriella Pinto, Maria Chiara Di Meo, Gerardo Alvarez-Rivera, Maria Michela Corsaro, Angela Amoresano, Armando Zarrelli, Elena Ibáñez, Antonello Merlino, Daria Maria Monti

**Affiliations:** † Department of Chemical Sciences, University of Naples Federico II, via Cinthia 4, Naples80126, Italy; ‡ Department of Sciences and Technologies (DST), University of Sannio, Benevento82100, Italy; § Laboratory of Foodomics, Institute of Food Science Research, CIAL, CSIC, Nicolás Cabrera 9, Madrid28049, Spain

**Keywords:** microalgae, cascade approach, high-added-value molecules, phycoerythrin, sulfated exopolysaccharides

## Abstract

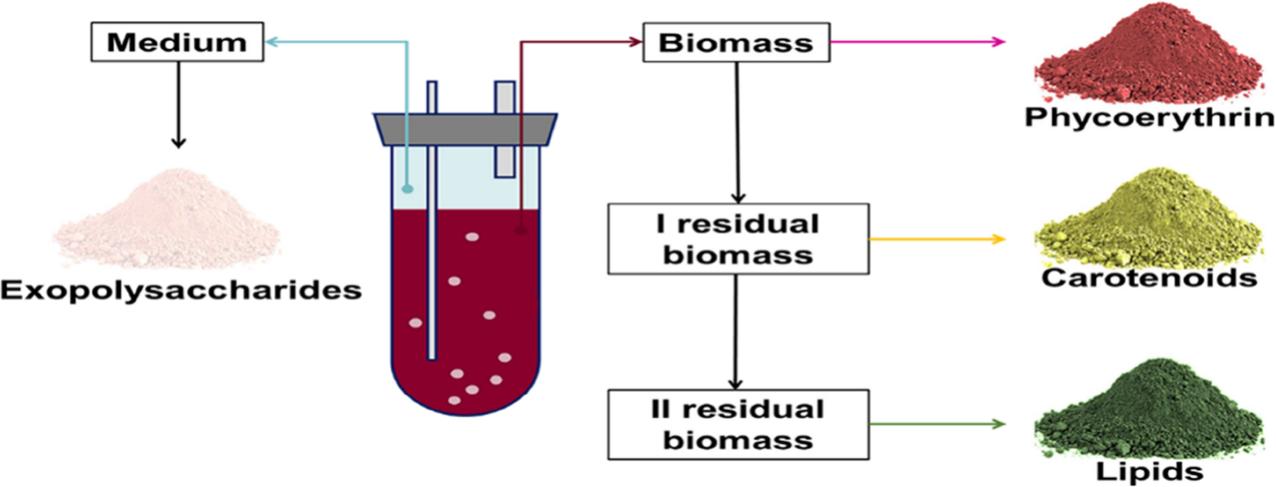

Here, an unprecedented biorefinery approach has been designed to recover high-added value bioproducts starting from the culture of*Porphyridium cruentum*. This unicellular marine red alga can secrete and accumulate high-value compounds that can find applications in a wide variety of industrial fields. 300 ± 67 mg/L of exopolysaccharides were obtained from cell culture medium; phycoerythrin was efficiently extracted (40% of total extract) and isolated by single chromatography, with a purity grade that allowed the crystal structure determination at 1.60 Å; a twofold increase in β-carotene yield was obtained from the residual biomass; the final residual biomass was found to be enriched in saturated fatty acids. Thus, for the first time, a complete exploitation of*P. cruentum*culture was set up.

## Abstract 2

A new concept in biorefinery:*Porphyridium cruentum*is fully exploited to recover high-added-value products.

## Introduction

Microalgae capture carbon dioxide during their growth to perform photosynthesis. This implies the production of oxygen and the reduction of carbon dioxide emissions.^1^ It is noteworthy that microalgae are used as a reliable source of food and high-added value products.^2^ Microalgae can be considered perfect candidates for their use in biorefinery approaches,^3^ as they can grow in lands which do not compete with food production, and with a higher growth rate with respect to conventional crops. From a theoretical point of view, a biorefinery is a combination of multiple integrated processes able to convert the biomass into a variety of high-added value products, in an economical and environmentally sustainable approach.^4^ This is in line with the principles of circular economy, fostered by international organizations and promoted by European Union (https://eur-lex.europa.eu/legal-content/EN/ALL/?uri=CELEX%3A52015DC0614). Unfortunately, in the microalgae field, a real biorefinery has been demonstrated only for few strains,^5^ and most of the present literature is focused on the extraction of one or two classes of molecules, thus suggesting that the process is not economically feasible.

*Porphyridium cruentum* is a red marine microalga, reservoir of potentially high-added value molecules, such as carotenoids, sulfated exopolysaccharides (EPSs), B-phycoerythrin (PE), and lipids.^6−9^ Carotenoids are well-known antioxidants, also able to counteract many disorders, from type 2 diabetes, to degenerative diseases or cancer.^10^ Sulfated EPSs have a chemical structure which confer them peculiar rheological properties^11^ and many biological activities,^12^ such as antimicrobial, anti-inflammatory,^13^ hypocholesterolemic,^14^ antiviral activities,^15^ and skin protective activity.^16^ PE, the main protein found in*P. cruentum*, has a good market value for different reasons: (i) in biomedical and molecular applications for its natural fluorescence; (ii) as a natural red-colored protein to be used as a dye for food and cosmetics, and (iii) in the pharmaceutical industry thanks to its antioxidant activity.^17^ Finally, recent literature exploited the use of saturated fatty acids as antimicrobial agents and as drug delivery systems.^18,19^

Thus, taking advantage of the chemical composition of*P. cruentum*, here we propose a real biorefinery. For the first time, not only the biomass but also the exhausted medium was fully exploited to recover: EPSs from the medium and PE, carotenoids and lipids from the biomass. Molecules were extracted sequentially, starting from the most valuable one, without affecting the activity of the molecules in the residual biomass.

## Materials and Methods

### Reagents

All chemicals, solvents, and reagents, unless differently specified, were from Sigma-Aldrich (St Louis, MO, USA).

### Microalgal Strain and Dry Weight Determination

*Porphyridium cruentum* strain was acquired from Culture Collection of Autotrophic Organism (CCALA, Centre for Phycology, Institute of Botany of the AS CR, Dukelská 135, TŘEBOŇ CZ-379 82, Czech Republic). Preculture (50 mL, 0.09 ± 0.01 O.D./mL) of *P. cruentum* was inoculated in *Porphyridium* medium^20^ in a 1 L bubble column photobioreactor (working volume 800 mL) in a room with constant temperature (25 ± 1 °C) and light (fluorescent lamps with an intensity of 13 ± 1 PAR ) in autotrophic conditions, without CO_2_. The culture was mixed by bubbling through a sintered glass tube placed at the bottom of each culture tube. Algal growth was monitored by measuring the absorbance at 730 nm. The O.D. values were converted into biomass amount by correlating O.D. and dry cell weight.

### Exopolysaccharide Isolation and Quantization

To process a high volume of medium, the latter was concentrated 10 times by lyophilization, prior to addition of pure ethanol (1:2 v/v); EPSs were then recovered by centrifugation (12,000 *g*, 30 min, 4 °C), collected and lyophilized. Total carbohydrates were quantified by the phenol–sulfuric acid method, according to Geresh et al.,^21^ with some modifications, as reported in Gallego.^4^ A reference curve was obtained by using glucose (0.03–1.0 mg/mL).

### Determination of Monosaccharide Composition

Lyophilized EPSs (2 mg) were solubilized with 1 mL of HCl/CH_3_OH (1.25 M) for 16 h at 80 °C.^22^ Then, the sample was dried and acetylated with 25 μL of acetic anhydride and 25 μL of pyridine and kept at 100 °C for 30 min. The mixture was analyzed by gas chromatography–mass spectrometry (GC–MS) by using an Agilent Technologies instrument (GC 7820A, MS 5977B), equipped with a HP-5MS 30 m, 0.25 mm, 0.25 μm capillary column. The following temperature program was used to analyze acetylated methyl glycosides: 140 °C for 3 min, 140 °C → 240 °C at 3 °C/min.

### Total Carbon, Hydrogen, Nitrogen, and Sulfur

The determination of total carbon (C), hydrogen (H), nitrogen (N), and sulfur (S) in EPSs recovered from *P. cruentum* medium was carried out by performing total combustion, using the FlashSmart Elemental Analyzer, according to Álvarez-Gómez and colleagues.^23^ Elements (C, H, N, S) were expressed as % with respect to the sample’s total weight.

### Protein Extraction and Quantification

Fresh biomass was harvested by centrifugation at 1200*g* for 30 min at room temperature and resuspended at 10 mg_D.W._/mL in PBS pH 7.4. Cell disruption was done by: (i) maceration; (ii) freeze and thaw; (iii) French Press, and (iv) sonication. For the maceration, the biomass was kept at 4 °C in agitation for 24 h. For the freeze and thaw method, the biomass was frozen (−80 °C) and then thawed (37 °C) for five cycles. For the French Press, two cycles were performed at a pressure of 2 kbar. The ultrasound method was performed by operating with MS73 tip at 40% amplitude of the instrument (Bandelin Sonoplus HD 3200) for different lengths of time, from 4 to 20 min, (30″ on, 30″ off), on ice. At the end of each step, samples were centrifuged at 5000 *g* at 4 °C for 30 min, proteins were recovered in the supernatant, total proteins were determined by BCA Protein Assay Kit (Thermo Scientific) and then SDS-PAGE analysis followed by Coomassie staining was performed. Phycobiliprotein concentration was determined by the Bennet and Bogorad equations:^24^
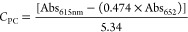
1
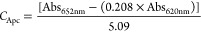
2
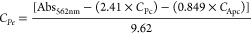
3

The reported wavelengths (562, 615, and 652 nm) correspond to the maximum of absorption of phycoerythrin, phycocyanin, and allophycocyanin, respectively.

### Phycoerythrin Purification

PE purification was performed by comparing three techniques: anion-exchange chromatography, gel filtration, and ultrafiltration. Anion-exchange chromatography was carried out by using a Nuvia-Q resin equilibrated with PBS pH 7.4, and elution was performed using 0.25 M NaCl. Gel filtration was performed by using a Sephadex G-75 equilibrated in PBS pH 7.4. Ultrafiltration was carried out with a 10 kDa molecular weight cut-off membrane, and the process was performed at 4 °C. The fractions obtained by anion-exchange and gel filtration, as well as the retentate obtained by ultrafiltration, were collected and analyzed by SDS-PAGE followed by Coomassie staining and PE purity grade was calculated by measuring the ratio Abs_562nm_/Abs_280nm_.

### Crystallization, Data Collection, Structure Solution, and Refinement of Phycoerythrin

PE crystals were grown by the hanging drop vapor diffusion method^25^ using a drop containing 10 mg/mL PE in 0.25 M ammonium sulfate, 25 mM potassium phosphate at pH 5.0, equilibrated with a reservoir containing 0.5 M ammonium sulfate, and 50 mM potassium phosphate at pH 5.0. Red crystals were visible after 1 week (Figure S1, Supporting Information).

The crystals were soaked in a cryoprotectant solution containing 30% (v/v) glycerol in the reservoir solution and cooled at −173 °C. Starting from one crystal, a high-resolution data set was collected at the XRD2 beamline at the Elettra synchrotron in Trieste, Italy, at −173 °C. Data were processed and scaled using Autoproc.^26^ The crystal was trigonal, space group R3, with unit cell parameters *a* = *b* = 186.59 Å, *c* = 59.19 Å, α = β = 90°, and γ = 120°. For data collection statistics, see Table S1 (Supporting Information). The structure of PE was solved by molecular replacement using PHASER,^27^ and the PE structure deposited in the Protein Data Bank under the accession 3V58,^28^ as a starting model. The structure shows the presence of two (αβ) dimers in the asymmetric unit. Visual inspection and model improvements were carried out using Coot.^29^ Refinements were carried out using Refmac 5.0.^30^*R* factor and *R* free values were used to optimize the refinement strategy. The final model, which has good geometries and refinement statistics (Table S1, Supporting Information), was deposited in the Protein Data Bank under the accession code 8B4N. Pymol (www.pymol.org) was used to obtain molecular-graphics figures.

### In Situ Digestion

A single PE crystal was solubilized in water and analyzed by SDS-PAGE. For in-gel hydrolysis, SDS-PAGE bands were excised from the gel lane, destained by consecutive cycles of 0.1 M NH_4_HCO_3_ at pH 8.0 and acetonitrile (ACN), followed by reduction (10 mM DTT in 100 mM NH_4_HCO_3_, 45 min, at 56 °C) and alkylation (55 mM IAM in 100 mM NH_4_HCO_3_, 30 min, at room temperature). The gel pieces were washed with 0.1 M NH_4_HCO_3_ of pH 8.0 and ACN and subjected to the enzymatic hydrolysis by covering them with 40 μL sequencing grade modified trypsin (10 ng/μL trypsin; 10 mM NH_4_HCO_3_) overnight at 37 °C. Peptide mixtures were eluted, vacuum-dried, and resuspended in 2% ACN acidified with 0.1% HCOOH. Tryptic peptide mixtures were analyzed by MALDI-TOF (AB SCIEX, Milan, Italy) to reveal the amino acid sequence of the three phycoerythrin chains.

### Mass Spectrometry Analyses

Matrix-assisted laser desorption/ionization (MALDI) mass spectrometry (MS) experiments were performed on a 5800 MALDI-TOF-TOF ABSciex equipped with a nitrogen laser (337 nm) (AB SCIEX, Milan, Italy). Starting from each band, aliquots of peptide mixture (0.5 μL) were mixed (1:1, v/v) with alphacyano hydroxycinnamic acid (10 mg/mL) in acetonitrile: 55 mM citric acid (70:30) solution. Calibration was done by using a calibration mixture from AB SCIEX (Monoisotopic (M + *n*H)^*n*+^: 904.46 Da des-Arg-Bradykinin, 1296.68 Da Angiotensin I, 1570.67 Da Glu-Fibrinopeptide B, 2093.08 Da ACTH (clip 1–17), 2465.19 Da ACTH (clip 18–39), 3657.92 Da ACTH (clip 7–38)). Peptides were identified by MS spectra, acquired using a mass (*m/z*) range of 400–4000 Da. Peptide mass fingerprinting was performed by MS digest of homologue phycoerythrin sequences. MALDI MS experiments were performed on a 5800 MALDI-TOF-TOF ABSciex equipped with a nitrogen laser (337 nm) (AB SCIEX, Milan, Italy). The instrument operated with an accelerating voltage of 20 kV, a grid voltage at 66% of the source voltage, and a delay time at 200 ns. Laser power was set to 3500 V for the spectra acquisition. Each spectrum represents the sum of 10,000 laser pulses from randomly chosen positions on the same target place. The data were reported as monoisotopic masses.

### Carotenoid Extraction and Characterization

Carotenoids were extracted from the dry biomass, either raw or after protein extraction. Extractions were performed in ethanol, as reported by Aremu, with some modifications.^31^ Briefly, for each extraction, 200 mg of freeze-dried biomass was suspended in pure ethanol (4 mL) and sonicated (40% amplitude, 4 min on ice, Bandelin Sonoplus HD 3200, tip MS73). The mixture volume was then adjusted to 20 mL and shaken for 24 h at 250 rpm in a dark room at 4 °C. The supernatant was collected by centrifugation at 12,000*g* for 10 min, dried under nitrogen stream, and then stored at −20 °C. Carotenoid identification was performed by HPLC-DAD-APCI-QTOF-MS/MS, whereas quantization was done by calibration curves obtained by using commercial zeaxanthin and β-carotene, according to a method previously described,^5^ with some modifications. The analysis of the extracts was carried out in an Agilent 1290 UHPLC system (Ultrahigh Performance Liquid Chromatography) equipped with a diode-array detector (DAD), coupled to an Agilent 6540 quadrupole-time-of-flight mass spectrometer (q-TOF MS) equipped with an atmospheric pressure chemical ionization (APCI) source, all from Agilent Technologies (Santa Clara, CA, USA). Extracts were solubilized in ethanol (5 mg/mL), filtered through 0.45 μm nylon filters, and then analyzed under positive ionization mode, using the following parameters: capillary voltage, 3.5 kV; drying temperature, 350 °C; vaporizer temperature, 400 °C; drying gas flow rate, 8 L/min; nebulizer gas pressure, 40 psi; corona current (which sets the discharge amperage for the APCI source), 4000 nA. The mass spectrometer was operated in MS and tandem MS modes for the structural analysis of all compounds. The MS and Auto MS/MS modes were set to acquire *m/z* values ranging between 50 and 1100 and 50 and 800, respectively, at a scan rate of 5 spectra per second.

### Lipids Extraction and Characterization

Lipids were extracted on the dried biomass. Both the raw and the two residual biomasses (I and II residual biomass) were dried at 60 °C for 24 h. Lipids were obtained as reported by Bligh and Dyer.^32^ Nitrogen flux was used to dry, recover, and weigh lipids. Lipid fractionation was performed by solubilizing them in chloroform and using a commercial prepacked column containing a stationary phase made of Florisil. Neutral lipids were eluted with chloroform:methanol (2:1, v/v), fatty acids with 2% acetic acid in diethyl-ether, whereas phospholipids were eluted with 100% methanol. The recovered fatty acids were then characterized by GC–MS, as previously reported.^33^

### Statistical Analyses

Results are reported as mean of results obtained after three independent experiments (mean ± SD) and compared by one-way analysis of variance according to Bonferroni’s method (post hoc) using Graphpad Prism for Windows, version 6.01.

## Results and Discussion

### Exopolysaccharide Recovery and Characterization

**Figure 1:**
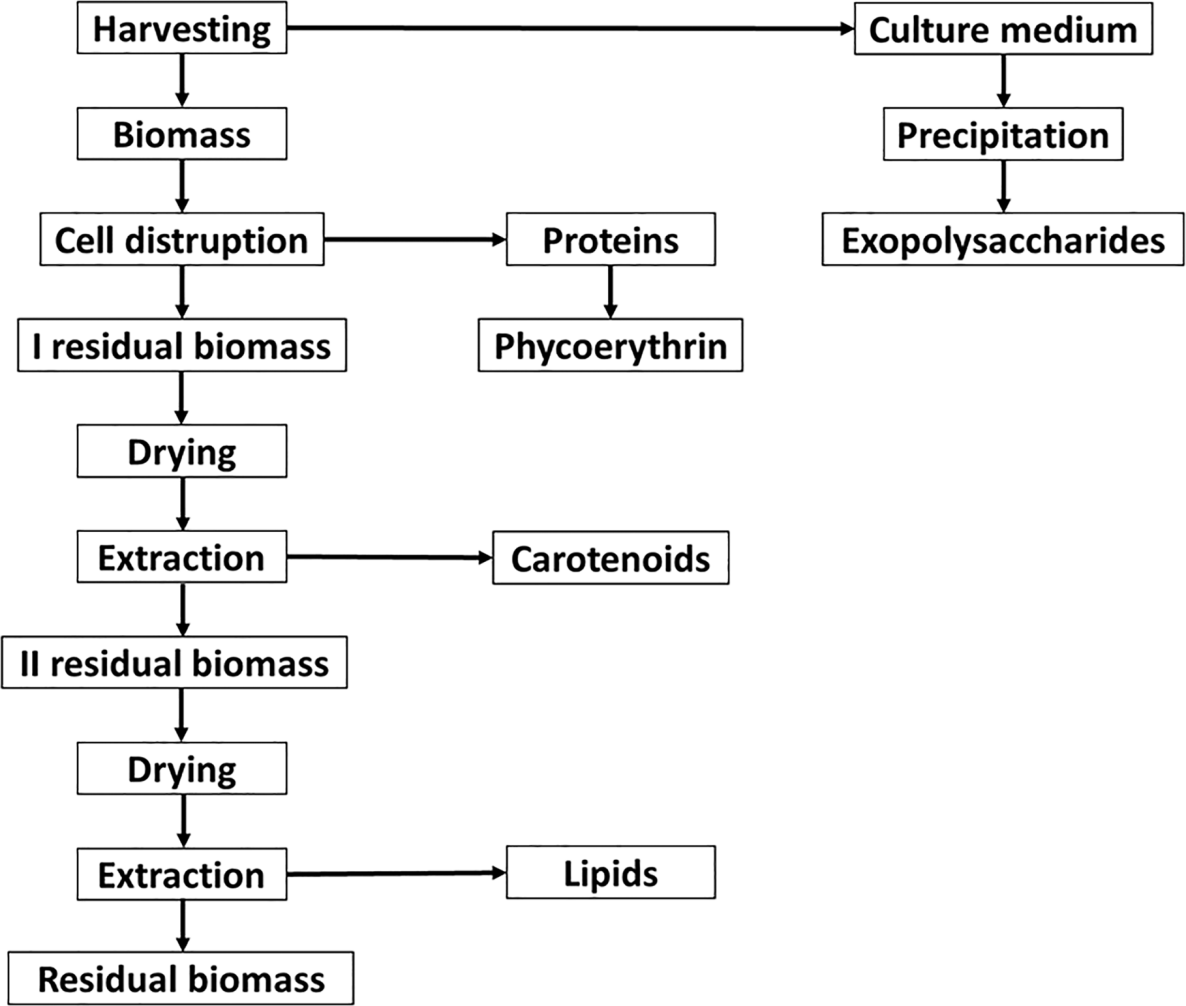
Schematic representation of the applied process.

**Figure 2:**
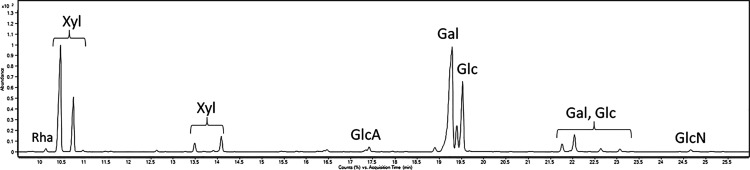
GC–MS chromatogram of the AMG of *P. cruentum* exopolysaccharides recovered from the culture medium. In the graph, the relative ion current abundance is reported as a function of retention time (min).

Figure 1 reports the extraction strategy used to recover different molecules from *P. cruentum*. At the end of cell growth, medium, generally regarded as waste, was collected and polysaccharides were isolated by precipitation using ethanol or 2-propanol. The EPS content was measured by the phenol–sulfuric acid method and no difference between the two applied solvents was observed (ethanol yield 0.100 ± 0.020 and 2-propanol 0.116 ± 0.020 mg/mL); thus ethanol was selected because of its wide range of applications.^34^ The EPS yield was found to be 300 ± 67 mg/L of culture medium, which corresponds to an EPS yield of 0.53 g/g_d.w.biomass_. The monosaccharide composition was achieved by GC–MS analysis, after derivatization as acetylated methyl glycosides (AMGs). The GC–MS chromatogram disclosed the presence of mainly xylose (Xyl), galactose (Gal), and glucose (Glc). Finally, traces of rhamnose (Rha), glucuronic acid (GlcA), and glucosamine (GlcN) were detected (Figure 2).

Because the EPSs from *P. cruentum* are known to be sulfated, the elemental composition analysis was performed. Results, reported in Table 1, indicate that the % of sulfur present in the sample is similar to that reported in the literature (7.4% ± 0.2%).^35,36^ No proteins were detected.

**Table 1:** Elemental Composition and Protein Content of EPS^a^

O (%)	N (%)	H (%)	C (%)	S (%)	protein content (mg/mL)
76.0 ± 2.4	1.1 ± 0.6	2.6 ± 0.4	13.1 ± 1.3	7.4 ± 0.2	N.D.

a Elements were measured by an elemental analyzer. Oxygen % was calculated by subtracting the % of the other elements from 100%. % are expressed as mg of each element/mg of analyzed EPS. Protein content was measured by colorimetric assay.

### Biomass Exploitation

#### Phycoerythrin Extraction Optimization

To extract PE, different extraction procedures were evaluated, as described in Materials and Methods. Total protein concentration was determined by colorimetric assay (BCA), whereas PE, phycocyanin (PC), and allophycocyanin (APC) concentrations were obtained using the Bennet and Bogorad equations (reported in the Material and Methods section). As shown in Table 2, the amount of PE was similar in all the analyzed samples, whereas PC and APC were found to be most abundant only in the extract obtained by French Press. Indeed, the presence of APC and PC in the French Press extract halved the purity grade of the extract with respect to those obtained for the other three extracts.

**Table 2:** Total Protein and Phycobiliprotein Concentration, Extraction Yield, and Phycoerythrin Purity Grade Obtained for Each Extraction Protocol^a^

	maceration	sonication	freeze and thaw	French press
protein yield (%)	44 ± 5	35 ± 4	35 ± 3	37 ± 6
PE yield (%)	1.8 ± 0.1	1.8 ± 0.8	1.8 ± 0.5	1.4 ± 0.7
PE purity grade (Abs_562nm_/Abs_280nm_)	0.8 ± 0.1	0.9 ± 0.1	1.1 ± 0.1	0.4 ± 0.2
PC yield (%)	0.14 ± 0.02	0.48 ± 0.1	0.21 ± 0.1	1.4 ± 0.5
APC yield (%)	0.13 ± 0.1	0.2 ± 0.1	0.5 ± 0.02	1.1 ± 0.2

a Total protein concentration was obtained by BCA assay and total PE, PC, and APC concentration by Bennett and Bogorad equations. Yields are expressed as *g*_protein_/*g*_d.w.biomass_. The PE purity grade was calculated from the Abs_562nm_/Abs_280nm_ ratio.

Supernatants were also analyzed by SDS-PAGE followed by Coomassie staining (Figure S2A, Supporting Information) and UVA light exposure, taking advantage of chromophores present in PE (Figure S2B, Supporting Information). The analyses revealed two major bands, whose molecular weights corresponded to α and β subunits (17 kDa) and γ subunit (30 kDa) of PE, in each lane. Results suggested ultrasound as the most promising method. As biomass storage is a crucial step in industrial-scale processes, also from a logistically point of view,^37^ extractions were performed by ultrasound on either fresh or frozen biomass (stored at −80 °C) for different lengths of time (from 4 to 20 min). At the end of each extraction, the disrupted biomass was analyzed by SDS-PAGE (Figure S2C,D, Supporting Information). Supernatants obtained after 20 min extraction seemed to be the best choice for fresh biomass, as 4 min extraction allowed to recover a PE content of 1.8% (Table 2) with a purity grade of 0.9 (Table 2), whereas 20 min of sonication on fresh biomass allowed to recover a PE content of 3.0 ± 0.4% with a purity grade of 1.5 ± 0.3. These extraction values are higher with respect to those obtained, after 20 min of sonication, from the frozen biomass: PE content of 2.2 ± 0.2 to 3.0 ± 0.4% for frozen and fresh biomass, respectively, and a PE purity grade of 1.0 ± 0.2 and 1.5 ± 0.3 for frozen and fresh biomass, respectively.

#### Phycoerythrin Purification and Structure Determination

**Figure 3:**
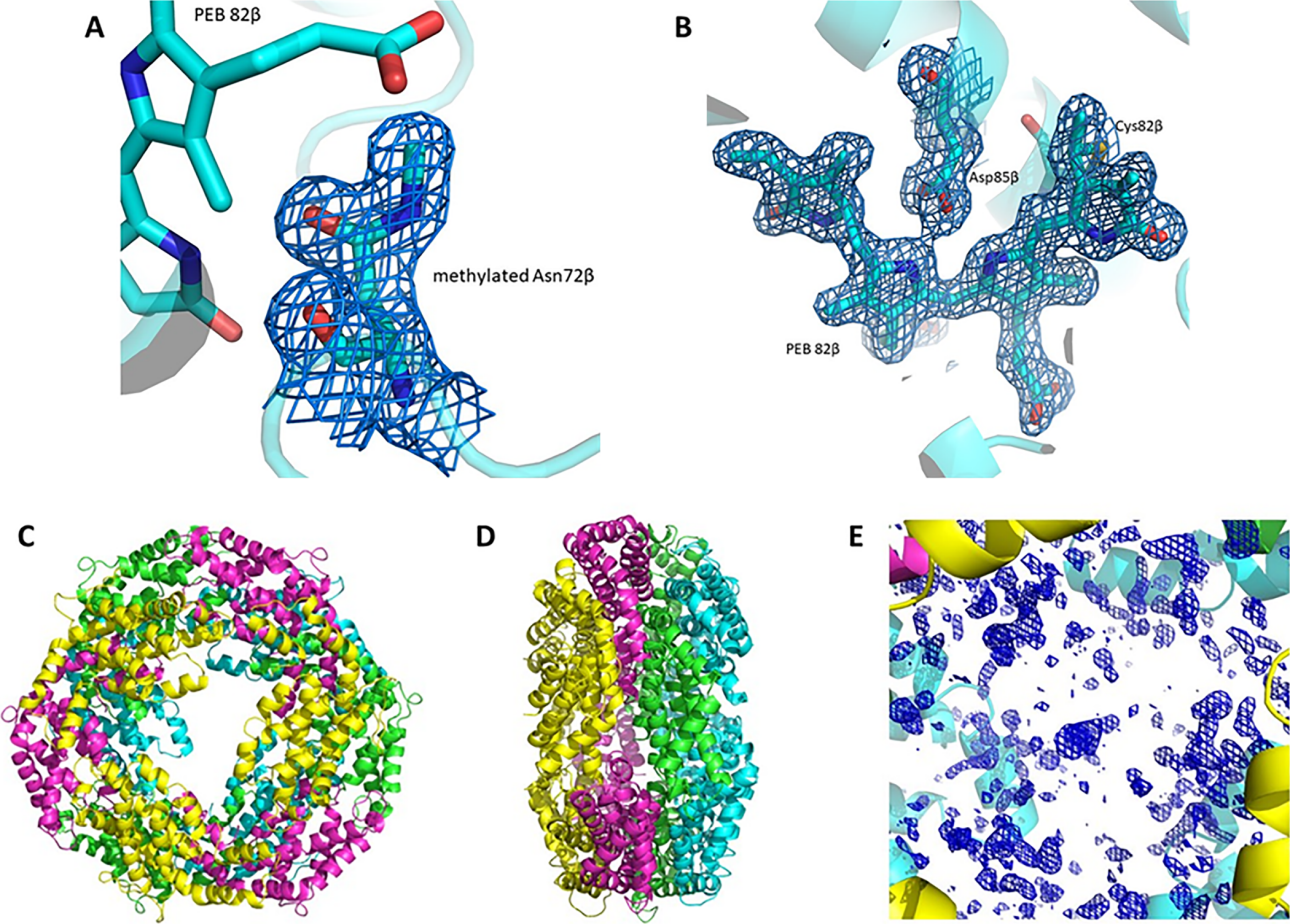
PE crystal structure. (A) 2Fo-Fc electron density map (1.0σ) of the methylated Asn72β in the structure of PE. (B) 2Fo-Fc electron density maps contoured at 1.0σ of one of the five PEB chromophores found in the structure of PE. (C) (αβ)_3_ hexamer shown from the upper view and the lateral view (D). Electron density of the central cavity is shown in panel E. This segment of electron density should contain information on the location of the γ subunit.

**Figure 4:**
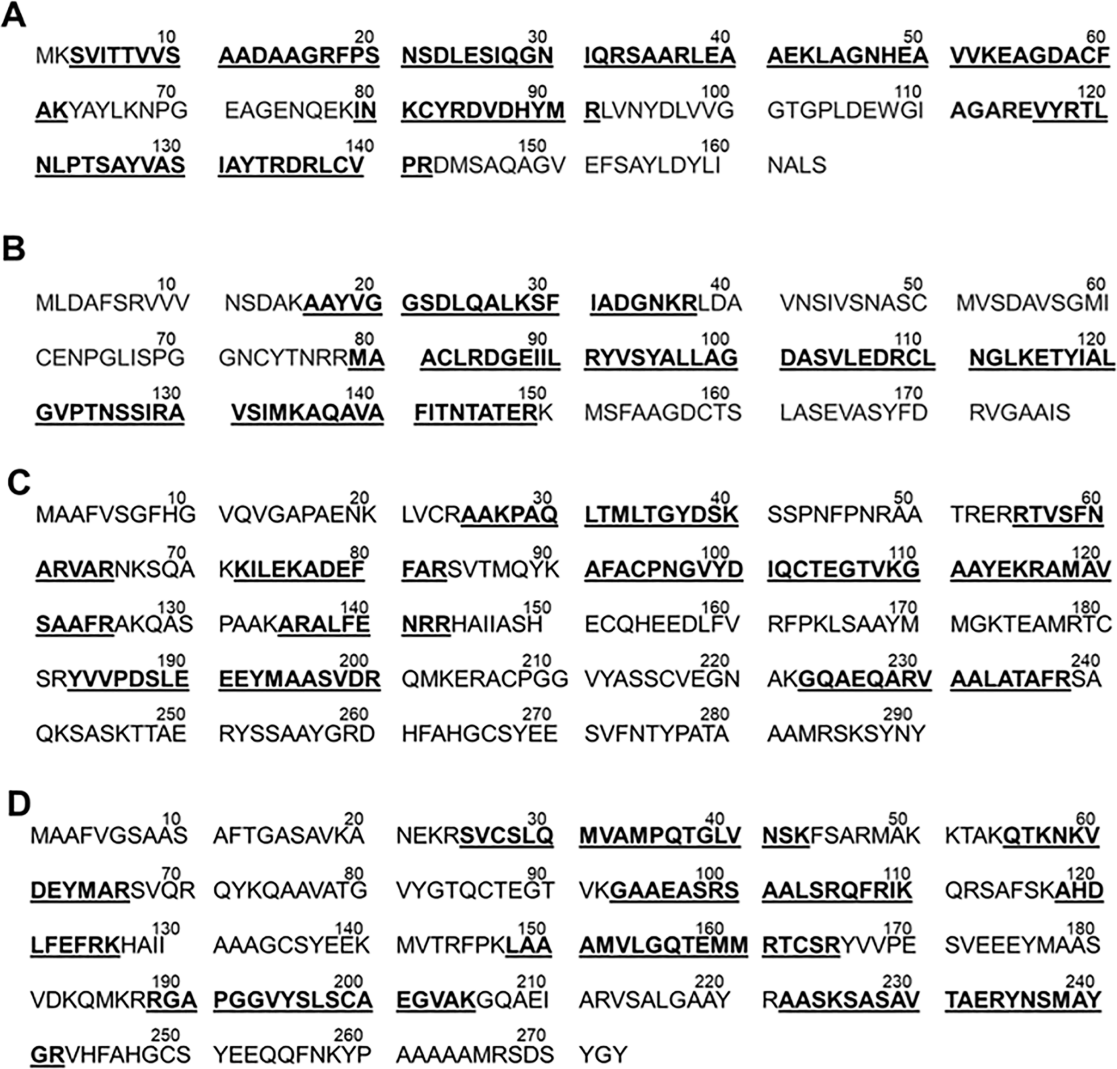
α, β, and γ chain sequences of *P. cruentum* detected by MALDI-TOF analysis. (A) α chain sequence displaying the different peptides (bold and underlined) compared to P11392 (PHEA_PORPP) sequence on UniProt. (B) β chain sequence displaying the different peptides (bold and underlined) compared to P11393 (PHEB_PORPP) sequence on UniProt. (C) γ chain sequence displaying the different peptides (bold and underlined) compared to A0A5J4YX19 (PHEB_PORPP) sequence on UniProt. (D) γ chain sequence displaying the different peptides (bold and underlined) compared to A0A5J4YZM7 (PHEB_PORPP) sequence on UniProt.

**Figure 5:**
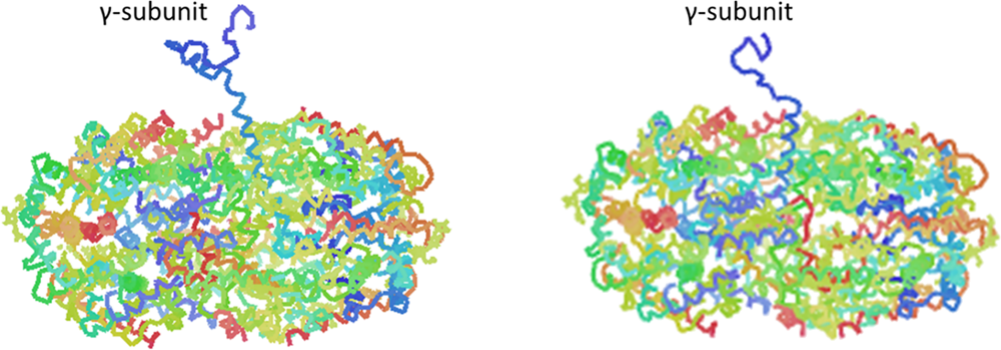
Heptameric (αβ)_3_γ structures observed in the phycobilisome from *P. cruentum*.

PE purification was performed by comparing three techniques: anion-exchange chromatography, gel filtration, and ultrafiltration. The results of the different techniques are reported in the Supporting Information (Figure S2E–G, Supporting Information). Only the size-exclusion chromatography allowed obtaining pure PE; in particular, this purification step allowed to recover about 80% of PE and to reach a purity grade of 4.0. It is known that a purity grade ≥4.0 indicates a protein to be used in analytical grade.^38^ According to the overall results, this approach allowed obtaining high-purity grade PE by only one step extraction and purification. To the best of our knowledge, this is the first time that PE was extracted and purified with such a purity grade by using only a single purification step.^39−42^ To determine the protein identity and purity, PE was crystallized, and its X-ray structure was determined at 1.60 Å resolution. The X-ray structure is constituted by 5990 atoms, including five phycoerythrobilin (PEB) chromophores for each αβ dimer, one methylated Asn in position β72 (Figure 3A) for each β subunit, two sulfate ions, and 324 water molecules and refines to *R* factor/*R*free values of 0.222/0.256. The structure confirms the formation of the (αβ)_3_ hexamer (Figure 3C,D) but does not allow to identify the exact location of the γ subunit (Figure 3E). Similar results were obtained in previous studies^43,44^ and were attributed to rotational disorder of the γ subunit within the protein crystal and to the finding that the electron density of this subunit is averaged out by the threefold crystallographic symmetry. The α subunit contains 164 residues, the β subunit 177 residues. The overall structure of the protein is very similar to that previously reported and deposited in the Protein Data Bank under accession code 3V58, obtained from crystals grown under different experimental conditions, but at the same pH. After superposition, the 164 CA atoms of the α subunit have a root-mean-square (r.m.s.) deviation of 0.146 Å, and the 177 CA atoms of β subunit have an r.m.s. deviation of 0.142 Å. Each αβ dimer has five PEBs in position α82, α139, β61, β82, and β158. The first PEB, which adopts two alternate conformations in our structure, is covalently attached to the side chain of Cys82 of the α subunit by ring A. The second PEB is bound to Cys139 of the same subunit. The other three PEBs are found in the β subunit. They are bound to the side chains of Cys61, Cys82, and Cys158. The stereochemistry of the chromophores and their interaction with protein residues are basically identical to that observed in the starting model^28^ and previously described. An example of the well-defined electron density maps of the chromophores is reported in Figure 3B. To verify the presence of a γ subunit, PE crystals were dissolved and analyzed by UV–vis absorption spectroscopy, SDS-PAGE analyses, and mass spectrometry. The UV–vis absorption spectrum (Figure S3A, Supporting Information) showed a peculiar shoulder at 498 nm, ascribed to the phycourobilin chromophore of the γ subunit. SDS-PAGE analysis (Figure S3B, Supporting Information) showed the presence of two molecular species, whose molecular mass was compatible with α and β subunits (double bands at about 17 kDa) and with the γ subunit (30 kDa). In situ digestion was performed on the bands and the peptide mixtures were analyzed by MALDI-TOF; peptide mass fingerprinting was performed by MS digest of homologue phycoerythrin sequences. The assignment of each mass spectrometry signal allowed highlighting the peptide sequence along the entire protein sequence. As shown in Figure 4,BA, the MS analysis of lower band enabled tracing the peptide sequence (labeled in bold and underlined) for the α and β chains, displaying a sequence coverage of 64 and 61%, respectively. The MS analysis of the higher band, on the other hand, revealed that the signals were attributed to two γ-chains with comparable sequence coverage (59.3 and 43.5%), as shown in Figure 4C,D. The presence of the two γ subunits has been previously found in the structure of the entire phycobilisome solved by electron microscopy (PDB code 6KGX).^45^ The heptameric structures of PE, with the two different γ subunits, extracted from PDB code 6KGX are reported in Figure 5, with the same orientation.

#### Carotenoid Extraction and Characterization

**Figure 6:**
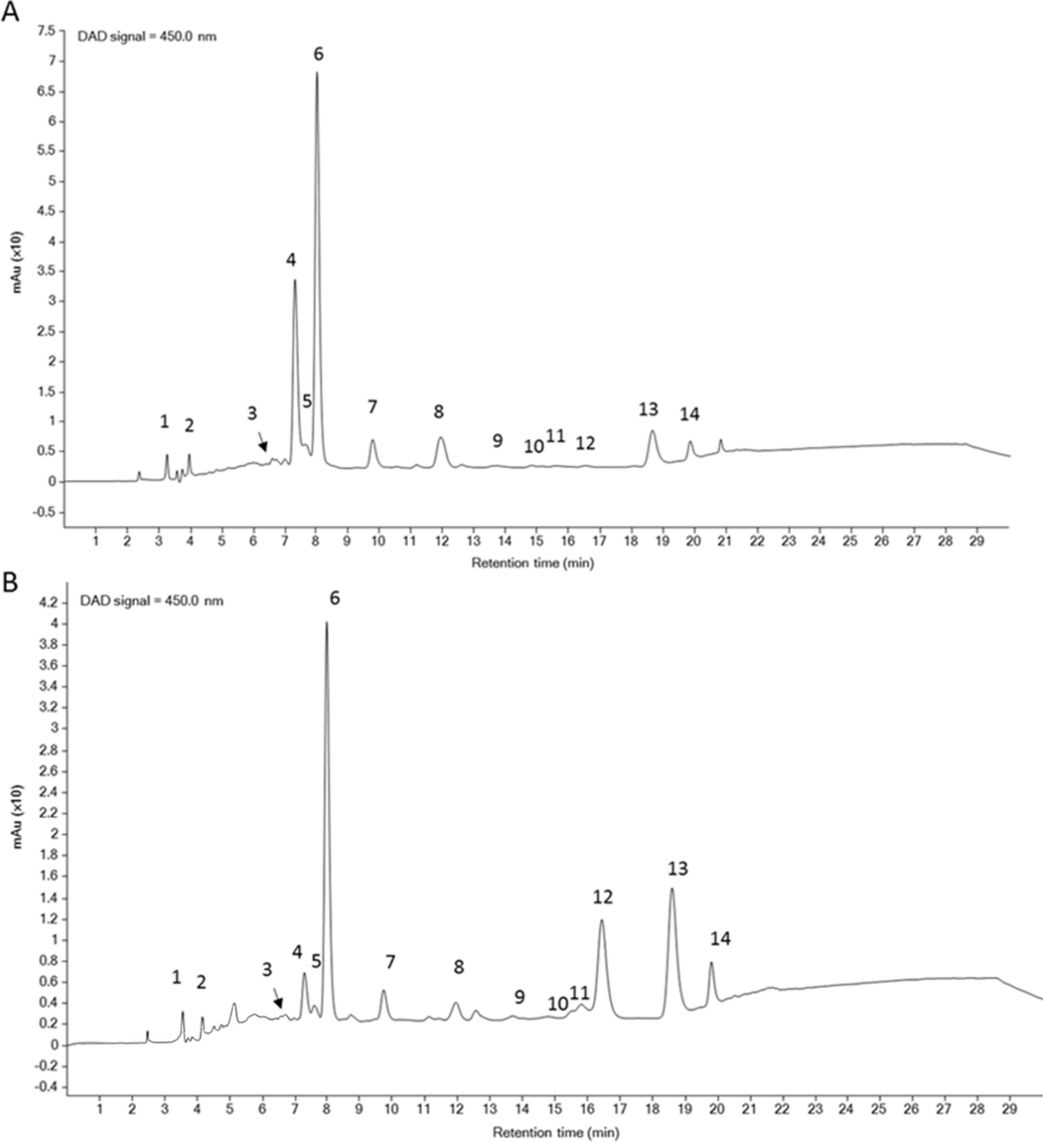
Representative HPLC–DAD chromatograms of carotenoids extracted from *P. cruentum* biomass. (A) Raw biomass; (B) I residual biomass (after PE extraction). Peak numbers and their identification are reported in Table S2, Supporting Information.

Carotenoid extraction was performed by using a conventional method on both residual biomass (herein referred as I residual biomass) and on the raw one, used as benchmark (as reported in the Materials and Methods section). HPLC analyses (Figure 6) revealed that zeaxanthin and β-carotene were the major pigments present in the two extracts. Quantification analyses for the two carotenoids are reported in Table 3 and indicate that all the zeaxanthin isoforms present in the I residual biomass represented about 55% of those present in the raw biomass, whereas β-carotene isoform recovery was about 210% with respect to the raw biomass. It is important to point out that the carotenoids yield obtained from the I residual biomass was comparable to the one obtained by innovative green extractions performed on the residual biomass of the same species,^4^ thus suggesting the feasibility of the proposed process.

**Table 3:** Comparison between Zeaxanthin, β-Carotene, and Their Isomers in the Raw and I Residual Extracts

peak number	retention time (min)	peak identification	raw biomass (mg/g_extract_)	I residual biomass (mg/g_extract_)
5	7.668	zeaxanthin isomer I	1.03	0.64
6	8.057	zeaxanthin	21.37	11.79
7	9.826	zeaxanthin isomer II	2.84	1.69
13	18.701	β-carotene	0.20	0.44
14	18.892	β-carotene isomer	0.04	0.07

### Lipid Extraction and Characterization

According to the strategy described in Figure 1, lipid extraction represented the last class of the proposed process, after a drying step. To verify if the previous extractions could affect lipid composition, control experiments were performed by determining the composition of the lipid fraction obtained after PE extraction (I residual biomass) and after PE and carotenoid extraction (II residual biomass). The extractions were performed according to Bligh and Dyer,^32^ followed by a solid phase extraction (SPE). As shown in Table 4, a 14% yield of total lipids was obtained from the raw biomass, whereas about 29% were extracted from the I residual biomass (i.e., after protein extraction). Nevertheless, when lipids were extracted from the II residual biomass (i.e., after protein and carotenoid extractions), the yield (14.1%) was comparable to those calculated for the raw biomass. Interestingly, the gas chromatography analysis of the fatty acid fraction revealed a clear trend: while polyunsaturated fatty acids (PUFAs) decreased during the cascade extractions (from 26 to 14 to 4%), a clear increase in the yield of saturated fatty acids (SFAs) was observed (from 72 to 76 to 93%). SFAs are considered now as an interesting new class of molecules, thanks to their chemical stability, responsible for their well-defined melting points and biocompatibility. Thus, they have been proposed as new materials for drug-controlled release or simply as antibacterial molecules.^18,19^

**Table 4:** Total Lipid Yields^a^

	lipid yield (%)	neutral lipids (%)	phospholipids (%)	fatty acids (%)	PUFA (%)	SFA (%)
raw biomass	14.0 ± 2.6	11.2 ± 1.5	12.0 ± 4.0	3.7 ± 0.3	25.8 ± 4.6	71.7 ± 13.6
I residual biomass	28.8 ± 1.5	10.2 ± 0.3	10.6 ± 0.3	2.5 ± 0.2	14.4 ± 1.9	76.3 ± 5.8
II residual biomass	14.1 ± 0.4	9.1 ± 0.6	3.8 ± 0.4	1.6 ± 0.2	4.3 ± 0.8	92.8 ± 7.2

a Lipids mean yields are reported as the percentage of the ratio between each lipidic class after SPE and dried raw biomass.

## Conclusions

The strategy proposed in Figure 1 was found to be effective, because an innovative and reliable strategy was set up to sequentially recover high-added-value products from *P. cruentum* culture. In particular, (i) *P. cruentum* culture is completely employed to recover intra- and extracellular class of high-added-value molecules; (ii) the yield of each extracted class of molecules is similar to those obtained when extractions are performed to recover single class of molecules; and (iii) according to the biorefinery principles, all the class of molecules have been recovered starting from the one with the highest market value. Simultaneous microalgae culture valorization and carbon capture can contribute to the sustainable expansion of the microalgae market.

## Supporting Information Available

Crystallographic data; PE extraction and purification analyses; carotenoid identification (PDF)

## References

[ref1] Zhang S.; Liu Z. Advances in the Biological Fixation of Carbon Dioxide by Microalgae. J. Chem. Technol. Biotechnol. 2021, 96, 1475–1495. 10.1002/jctb.6714.

[ref2] Bhalamurugan G. L.; Valerie O.; Mark L. Valuable Bioproducts Obtained from Microalgal Biomass and Their Commercial Applications: A Review. Environ. Eng. Res. 2018, 23, 229–241. 10.4491/eer.2017.220.

[ref3] Yen H. W.; Hu I. C.; Chen C. Y.; Ho S. H.; Lee D. J.; Chang J. S. Microalgae-Based Biorefinery - From Biofuels to Natural Products. Bioresour. Technol. 2013, 135, 166–174. 10.1016/j.biortech.2012.10.099. 23206809

[ref4] Gallego R.; Martínez M.; Cifuentes A.; Ibáñez E.; Herrero M. Development of a Green Downstream Process for the Valorization of *Porphyridium Cruentum* Biomass. Molecules 2019, 24, 1564 10.3390/molecules24081564. 31009991 PMC6515528

[ref5] Imbimbo P.; Bueno M.; D’Elia L.; Pollio A.; Ibañez E.; Olivieri G.; Monti D. M. Green Compressed Fluid Technologies To Extract Antioxidants and Lipids from *Galdieria Phlegrea* in a Biorefinery Approach. ACS Sustainable Chem. Eng. 2020, 8, 2939–2947. 10.1021/acssuschemeng.9b07505. 33828932 PMC8016174

[ref6] Feller R.; Matos Â. P.; Mazzutti S.; Moecke E. H. S.; Tres M. V.; Derner R. B.; Oliveira J. V.; Junior A. F. Polyunsaturated ω-3 and ω-6 Fatty Acids, Total Carotenoids and Antioxidant Activity of Three Marine Microalgae Extracts Obtained by Supercritical CO_2_ and Subcritical N-Butane. J. Supercrit. Fluids 2018, 133, 437–443. 10.1016/J.SUPFLU.2017.11.015.

[ref7] Di Lena G.; Casini I.; Lucarini M.; Lombardi-Boccia G. Carotenoid Profiling of Five Microalgae Species from Large-Scale Production. Food Res. Int. 2019, 120, 810–818. 10.1016/J.FOODRES.2018.11.043. 31000301

[ref8] Rebolloso Fuentes M. M.; Acién Fernández G. G.; Sánchez Pérez J. A.; Guil Guerrero J. L. Biomass Nutrient Profiles of the Microalga *Porphyridium Cruentum*. Food Chem. 2000, 70, 345–353. 10.1016/S0308-8146(00)00101-1.

[ref9] Gaignard C.; Gargouch N.; Dubessay P.; Delattre C.; Pierre G.; Laroche C.; Fendri I.; Abdelkafi S.; Michaud P. New Horizons in Culture and Valorization of Red Microalgae. Biotechnol. Adv. 2019, 37, 193–222. 10.1016/J.BIOTECHADV.2018.11.014. 30500354

[ref10] Rodriguez-Concepcion M.; Avalos J.; Bonet M. L.; Boronat A.; Gomez-Gomez L.; Hornero-Mendez D.; Limon M. C.; Meléndez-Martínez A. J.; Olmedilla-Alonso B.; Palou A.; Ribot J.; Rodrigo M. J.; Zacarias L.; Zhu C. A Global Perspective on Carotenoids: Metabolism, Biotechnology, and Benefits for Nutrition and Health. Prog. Lipid Res. 2018, 70, 62–93. 10.1016/J.PLIPRES.2018.04.004. 29679619

[ref11] Xiao R.; Zheng Y. Overview of Microalgal Extracellular Polymeric Substances (EPS) and Their Applications. Biotechnol. Adv. 2016, 34, 1225–1244. 10.1016/J.BIOTECHADV.2016.08.004. 27576096

[ref12] De Jesus Raposo M. F.; De Morais R. M. S. C.; De Morais A. M. M. B. Bioactivity and Applications of Sulphated Polysaccharides from Marine Microalgae. Mar. Drugs 2013, 11, 233–252. 10.3390/md11010233. 23344113 PMC3564169

[ref13] Mišurcová L.; Škrovánková S.; Samek D.; Ambrožová J.; Machů L. Health Benefits of Algal Polysaccharides in Human Nutrition. Adv. Food Nutr. Res. 2012, 66, 75–145. 10.1016/B978-0-12-394597-6.00003-3. 22909979

[ref14] Dvir I.; Stark A. H.; Chayoth R.; Madar Z.; Arad S. M. Hypocholesterolemic Effects of Nutraceuticals Produced from the Red Microalga *Porphyridium Sp*. in Rats. Nutrients 2009, 1, 156–167. 10.3390/nu1020156. 22253975 PMC3257595

[ref15] Huheihel M.; Ishanu V.; Tal J.; Arad S. Activity of *Porphyridium Sp*. Polysaccharide against Herpes Simplex Viruses *in Vitro* and *in Vivo*. J. Biochem. Biophys. Methods 2002, 50, 189–200. 10.1016/S0165-022X(01)00186-5. 11741707

[ref16] Meléndez-Martínez A. J.; Mapelli-Brahm P.; Stinco C. M. The Colourless Carotenoids Phytoene and Phytofluene: From Dietary Sources to Their Usefulness for the Functional Foods and Nutricosmetics Industries. J. Food Compos. Anal. 2018, 67, 91–103. 10.1016/J.JFCA.2018.01.002.

[ref17] Qiu J.; Madoz-Gurpide J.; Misek D. E.; Kuick R.; Brenner D. E.; Michailidis G.; Haab B. B.; Omenn G. S.; Hanash S. Development of Natural Protein Microarrays for Diagnosing Cancer Based on an Antibody Response to Tumor Antigens. J. Proteome Res. 2004, 3, 261–267. 10.1021/pr049971u. 15113102

[ref18] Yoon B.; Jackman J.; Valle-González E.; Cho N. J. Antibacterial Free Fatty Acids and Monoglycerides: Biological Activities, Experimental Testing, and Therapeutic Applications. Int. J. Mol. Sci. 2018, 19, 1114 10.3390/ijms19041114. 29642500 PMC5979495

[ref19] Xue K.; Lv S.; Zhu C. Bringing Naturally-Occurring Saturated Fatty Acids into Biomedical Research. J. Mater. Chem. B 2021, 9, 6973–6987. 10.1039/d1tb00843a. 34047743

[ref20] Brody M.; Emerson R. The Effect of Wavelength and Intensity of Light on the Proportion of Pigments in *Porphyridium Cruentum*. Am. J. Bot. 1959, 46, 433–440. 10.1002/j.1537-2197.1959.tb07034.x.

[ref21] Geresh S.; Adin I.; Yarmolinsky E.; Karpasas M. Characterization of the Extracellular Polysaccharide of *Porphyridium Sp.*: Molecular Weight Determination and Rheological Properties. Carbohydr. Polym. 2002, 50, 183–189. 10.1016/S0144-8617(02)00019-X.

[ref22] Ricciardelli A.; Casillo A.; Vergara A.; Balasco N.; Corsaro M. M.; Tutino M. L.; Parrilli E. Environmental Conditions Shape the Biofilm of the Antarctic Bacterium *Pseudoalteromonas Haloplanktis* TAC125. Microbiol. Res. 2019, 218, 66–75. 10.1016/j.micres.2018.09.010. 30454660

[ref23] Álvarez-Gómez F.; Korbee N.; Casas-Arrojo V.; Abdala-Díaz R. T.; Figueroa F. L. UV Photoprotection, Cytotoxicity and Immunology Capacity of Red Algae Extracts. Molecules 2019, 24, 341 10.3390/molecules24020341. 30669361 PMC6359249

[ref24] Bennett A.; Bogobad L. Complementary chromatic adaptation in a filamentous blue-green alga. J. Cell Biol. 1973, 58, 419–435. 10.1083/JCB.58.2.419. 4199659 PMC2109051

[ref25] Russo Krauss I.; Merlino A.; Vergara A.; Sica F. An Overview of Biological Macromolecule Crystallization. Int. J. Mol. Sci. 2013, 14, 11643 10.3390/ijms140611643. 23727935 PMC3709751

[ref26] Vonrhein C.; Flensburg C.; Keller P.; Sharff A.; Smart O.; Paciorek W.; Womack T.; Bricogne G. Data Processing and Analysis with the autoPROC Toolbox. Acta Crystallogr. Sect. D Biol. Crystallogr. 2011, 67, 293–302. 10.1107/S0907444911007773. 21460447 PMC3069744

[ref27] McCoy A. J.; Grosse-Kunstleve R. W.; Adams P. D.; Winn M. D.; Storoni L. C.; Read R. J. Phaser Crystallographic Software. J. Appl. Crystallogr. 2007, 40, 658–674. 10.1107/S0021889807021206. 19461840 PMC2483472

[ref28] Camara-Artigas A.; Bacarizo J.; Andujar-Sanchez M.; Ortiz-Salmeron E.; Mesa-Valle C.; Cuadri C.; Martin-Garcia J. M.; Martinez-Rodriguez S.; Mazzuca-Sobczuk T.; Ibañez M. J.; Allen J. P. PH-Dependent Structural Conformations of B-Phycoerythrin from *Porphyridium Cruentum*. FEBS J. 2012, 279, 3680–3691. 10.1111/j.1742-4658.2012.08730.x. 22863205

[ref29] Emsley P.; Cowtan K. Coot: Model-Building Tools for Molecular Graphics. Acta Crystallogr. Sect. D Biol. Crystallogr. 2004, 60, 2126–2132. 10.1107/S0907444904019158. 15572765

[ref30] Murshudov G. N.; Vagin A. A.; Dodson E. J. Refinement of Macromolecular Structures by the Maximum-Likelihood Method. Acta Crystallogr. Sect. D Biol. Crystallogr. 1997, 53, 240–255. 10.1107/S0907444996012255. 15299926

[ref31] Aremu A. O.; Masondo N. A.; Molnár Z.; Stirk W. A.; Ördög V.; Van Staden J. Changes in Phytochemical Content and Pharmacological Activities of Three Chlorella Strains Grown in Different Nitrogen Conditions. J. Appl. Phycol. 2016, 28, 149–159. 10.1007/s10811-015-0568-7.

[ref32] Bligh E. G.; Dyer W. J. A Rapid Method of Total Lipid Extraction and Purification. Can. J. Biochem. Physiol. 1959, 37, 911–917. 10.1139/o59-099. 13671378

[ref33] Imbimbo P.; Romanucci V.; Pollio A.; Fontanarosa C.; Amoresano A.; Zarrelli A.; Olivieri G.; Monti D. M. A Cascade Extraction of Active Phycocyanin and Fatty Acids from *Galdieria Phlegrea*. Appl. Microbiol. Biotechnol. 2019, 9455–9464. 10.1007/s00253-019-10154-0. 31696285

[ref34] Melo T.; Figueiredo A. R. P.; da Costa E.; Couto D.; Silva J.; Domingues M. R.; Domingues P. Ethanol Extraction of Polar Lipids from *Nannochloropsis Oceanica* for Food, Feed, and Biotechnology Applications Evaluated Using Lipidomic Approaches. Mar. Drugs 2021, 19, 593 10.3390/md19110593. 34822464 PMC8624173

[ref35] Bernaerts T. M. M.; Kyomugasho C.; Van Looveren N.; Gheysen L.; Foubert I.; Hendrickx M. E.; Van Loey A. M. Molecular and Rheological Characterization of Different Cell Wall Fractions of *Porphyridium Cruentum*. Carbohydr. Polym. 2018, 195, 542–550. 10.1016/J.CARBPOL.2018.05.001. 29805010

[ref36] Ben Hlima H.; Smaoui S.; Barkallah M.; Elhadef K.; Tounsi L.; Michaud P.; Fendri I.; Abdelkafi S. Sulfated Exopolysaccharides from *Porphyridium Cruentum*: A Useful Strategy to Extend the Shelf Life of Minced Beef Meat. Int. J. Biol. Macromol. 2021, 193, 1215–1225. 10.1016/J.IJBIOMAC.2021.10.161. 34717983

[ref37] Gruber-Brunhumer M. R.; Jerney J.; Zohar E.; Nussbaumer M.; Hieger C.; Bromberger P.; Bochmann G.; Jirsa F.; Schagerl M.; Obbard J. P.; Fuchs W.; Drosg B. Associated Effects of Storage and Mechanical Pre-Treatments of Microalgae Biomass on Biomethane Yields in Anaerobic Digestion. Biomass Bioenergy 2016, 93, 259–268. 10.1016/J.BIOMBIOE.2016.07.013.

[ref38] Fernández-Rojas B.; Hernández-Juárez J.; Pedraza-Chaverri J. Nutraceutical Properties of Phycocyanin. J. Funct. Foods 2014, 11, 375–392. 10.1016/j.jff.2014.10.011.

[ref39] Nguyen H. P. T.; Morançais M.; Déléris P.; Fleurence J.; Nguyen-Le C. T.; Vo K. H.; Dumay J. Purification of R-Phycoerythrin from a Marine Macroalga *Gracilaria Gracilis* by Anion-Exchange Chromatography. J. Appl. Phycol. 2020, 32, 553–561. 10.1007/s10811-019-01947-x.

[ref40] Serucňik M.; Vicente F. A.; Brecǩo Ž.; Coutinho J. A. P.; Ventura S. P. M.; Žnidaršič-Plazl P. Development of a Microfluidic Platform for R-Phycoerythrin Purification Using an Aqueous Micellar Two-Phase System. ACS Sustainable Chem. Eng. 2020, 8, 17097–17105. 10.1021/ACSSUSCHEMENG.0C05042. 33344096 PMC7737240

[ref41] Vicente F. A.; Cardoso I. S.; Martins M.; Gonçalves C. V. M.; Dias A. C. R. V.; Domingues P.; Coutinho J. A. P.; Ventura S. P. M. R-Phycoerythrin Extraction and Purification from Fresh *Gracilaria Sp*. Using Thermo-Responsive Systems. Green Chem. 2019, 21, 3816–3826. 10.1039/C9GC00104B.

[ref42] Ulagesan S.; Nam T. J.; Choi Y. H. Extraction and Purification of R-Phycoerythrin Alpha Subunit from the Marine Red Algae *Pyropia Yezoensis* and Its Biological Activities. Molecules 2021, 26, 6479 10.3390/MOLECULES26216479. 34770894 PMC8587297

[ref43] Ficner R.; Huber R. Refined Crystal Structure of Phycoerythrin from *Porphyridium Cruentum* at 0.23-Nm Resolution and Localization of the Gamma Subunit. Eur. J. Biochem. 1993, 218, 103–106. 10.1111/j.1432-1033.1993.tb18356.x. 8243457

[ref44] Ritter S.; Hiller R. G.; Wrench P. M.; Welte W.; Diederichs K. Crystal Structure of a Phycourobilin-Containing Phycoerythrin at 1.90-Å Resolution. J. Struct. Biol. 1999, 126, 86–97. 10.1006/jsbi.1999.4106. 10388620

[ref45] Ma J.; You X.; Sun S.; Wang X.; Qin S.; Sui S. F. Structural Basis of Energy Transfer in *Porphyridium Purpureum* Phycobilisome. Nature 2020, 579, 146–151. 10.1038/s41586-020-2020-7. 32076272

