# Supplementary material for: Inside out Porphyridium cruentum: Beyond the Conventional Biorefinery Concept
Source: ACS Sustain Chem Eng. 2022 Nov 29;11(1):381–9. doi: 10.1021/acssuschemeng.2c05869 (PMC9832536; doi:10.1021/acssuschemeng.2c05869)
Supplement: Supplementary file 1 — sc2c05869_si_001.pdf [file sc2c05869_si_001.pdf]

Inside out *Porphyridium cruentum*: beyond the conventional biorefinery concept

Davide Liberti[a], Paola Imbimbo[a], Enrica Giustino[a], Luigi D'Elia[a], Giarita Ferraro[a], Angela Casillo[a], Anna Illiano[a], Gabriella Pinto[a], Maria Chiara Di Meo[b], Gerardo Alvarez-Rivera[c], Maria Michela Corsaro[a], Angela Amoresano[a], Armando Zarrelli[a], Elena Ibanez[c], Antonello Merlino[a], Daria Maria Monti[a*]

[a] Department of Chemical Sciences, University of Naples Federico II, via Cinthia 4, 80126, Naples, Italy.

[b] Department of Sciences and Technologies (DST), University of Sannio, 82100 Benevento, Italy.

[c] Laboratory of Foodomics, Institute of Food Science Research, CIAL, CSIC, Nicolás Cabrera 9, 28049 Madrid, Spain.

*corresponding author: mdmonti@unina.it

Table of contents:

Number of pages: 7

Number of Tables: 2

Number of Figures: 3

Table S1. Data collection and refinement statistics.

Figure S1. PE crystals.

Figure S2. PE extraction and purification.

Figure S3. Analyses of PE dissolved crystals.

Table S2. Carotenoids peaks identification

Table S1. Data collection and refinement statistics.

Data collection	
Space group	R3
a=b, c (Å)	186.590, 59.190
α=β, γ (°)	90.0, 120.0
Molecules for asymmetric unit	Two αβ dimers
Observed reflections	925709 (44973)
Unique reflections	100909 (5012)
Resolution (Å)	46.65– 1.60 (1.62– 1.60)
Completeness (%)	99.0 (98.3)
Rmerge (%)	0.101 (1.028)
Average I/σ(I)	12.9 (2.2)
Multiplicity	9.2 (9.0)
$CC_{1\backslash 2}$	0.997 (0.595)
Refinement	
Resolution (Å)	46.65 -1.60
N° reflections	95441
N° reflections in working set	6986
Rfactor/Rfree	0.223/0.256
N° non-H atoms in the refinement	5990
B-factor overall ($Å^2$)	18.1
Average B-factor	23.93
Ramachandran values (%)	
Most favoured/ Additional allowed	97.19
Outliers	0.62
R.m.s.d. from ideality	
R.m.s.d. bonds (Å)	0.010
R.m.s.d. angles (°)	1.602

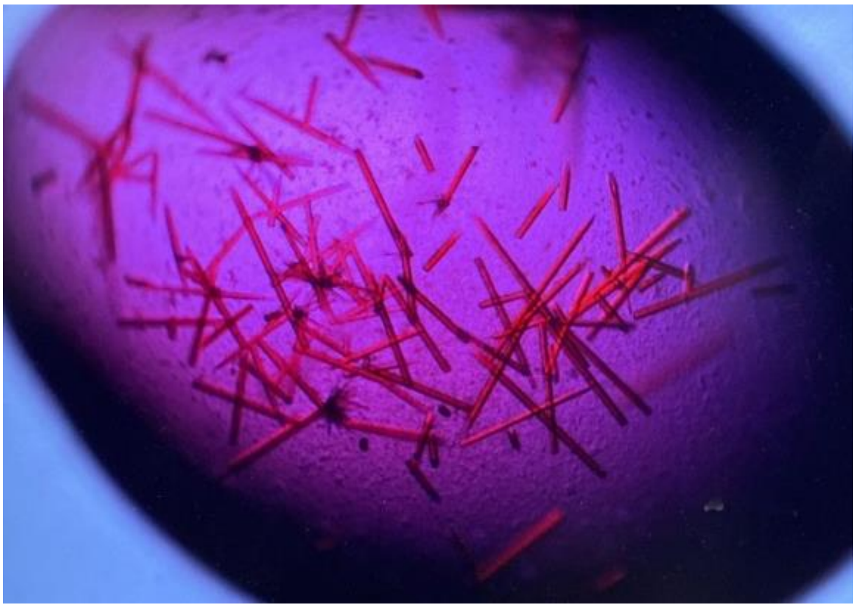

Figure S1. PE crystals. Crystals were grown by hanging drop vapor diffusion method using a drop containing 10 mg/mL PE in 0.250 M ammonium sulphate, 0.025 M potassium phosphate at pH 5.0, equilibrated with a reservoir containing 0.500 M ammonium sulphate, 0.050 M potassium phosphate at pH 5.0.

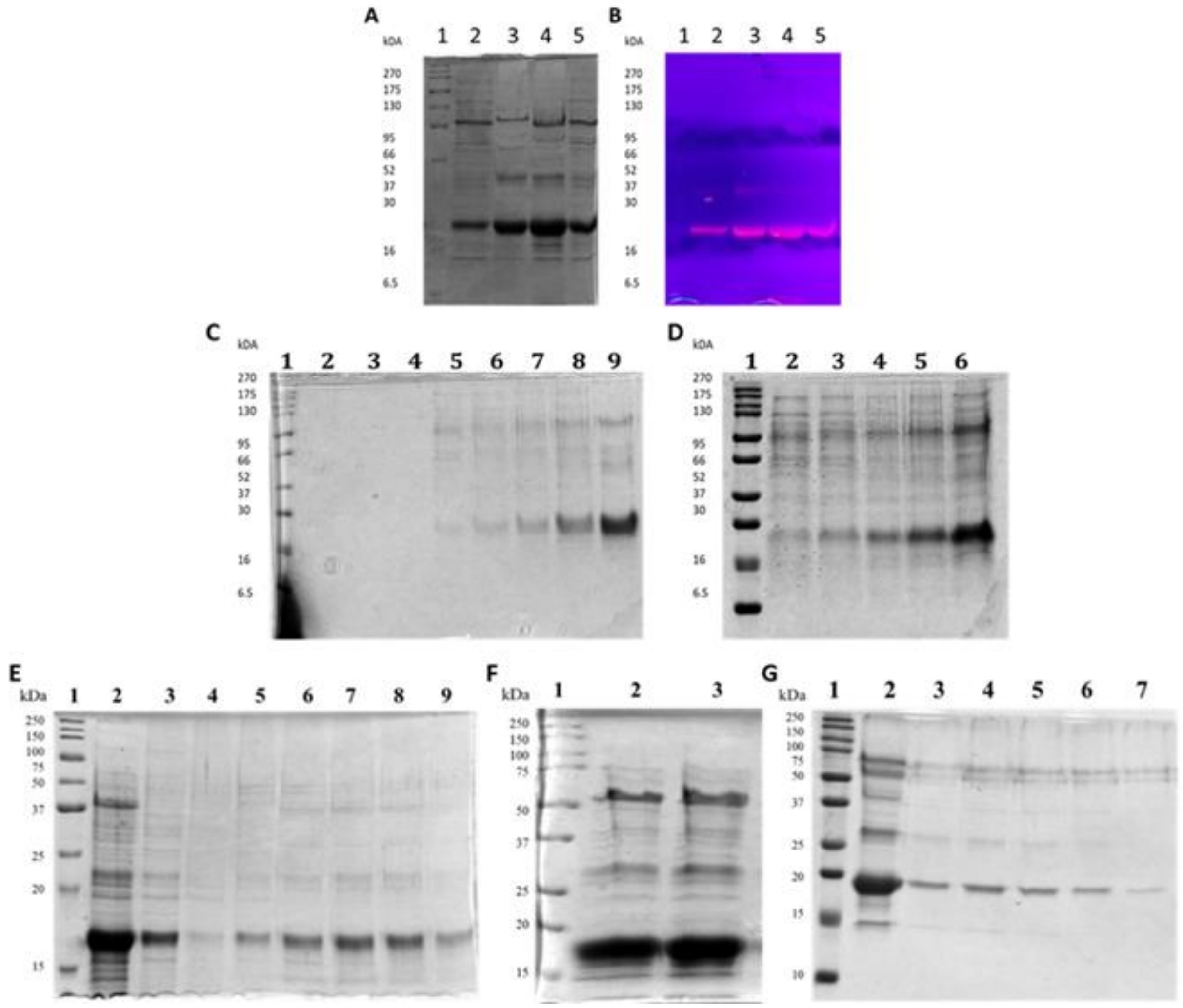

Figure S2. PE extraction and purification. Coomassie stained (**A**) and UVA light exposed unstained (**B**) SDS-PAGE analysis of total proteins extracted with different techniques. Lane 1: protein molecular weight markers; lane 2: Freeze & thaw extract; lane 3: Ultrasound extract; lane 4: Maceration extract; lane 5: French Press extract. 30 µg of total proteins were loaded in each lane. **C, D,** Coomassie staining of SDS-PAGE. Proteins were extracted by ultrasounds for different times. **(C)** fresh biomass and **(D)** frozen biomass. **C:** Lane 1: protein molecular weight markers; lanes 2-4: empty lanes; lanes 5-9: proteins extracted for 4, 8, 12, 16, and 20 minutes, respectively. **D:** Lane 1: protein molecular weight markers; lanes 2-6: proteins extracted for 4, 8, 12, 16, and 20 minutes, respectively. **E-G.** SDS-PAGE analysis of different procedures to isolate PE from *P. cruentum.* **E**: Anion-exchange. Lane 1: molecular weight markers; lane 2: total protein extract, lane 3: unbound; lane 4: washing fraction 1; lane 5: washing fraction 3; lane 6-9: samples eluted by 0.25 M NaCl. **F**: Ultrafiltration. Lane 1: molecular weight markers; lane 2: total protein extract; lane 3: ultrafiltration retentate. **G**: Size exclusion chromatography. Lane 1: molecular weight markers; lane 2: total protein extract; lanes 3-7: samples eluted from gel filtration. In all lanes, 30 µg of total proteins were analyzed.

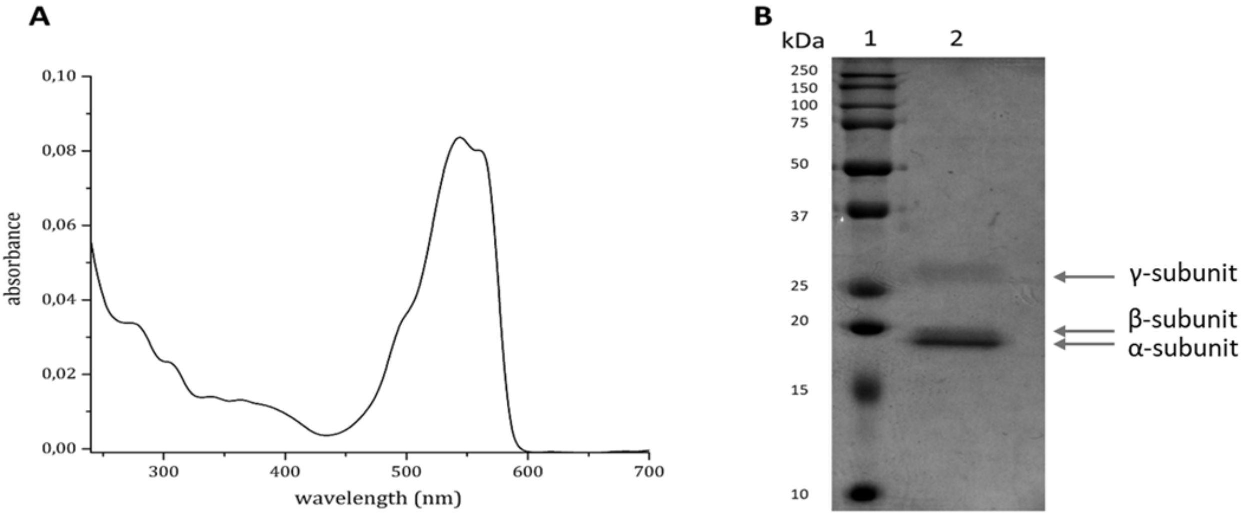

Figure S3. Analyses of PE dissolved crystals. A: UV-vis spectrum obtained from PE dissolved crystals. Spectrum was acquired at 25 °C, in the range 400–700 nm. **B**: SDS-PAGE analyses of PE dissolved crystals. Lane 1: molecular weight markers; lane 2: 20 μL of PE dissolved crystals.

Table S2. Tentatively identified compounds from *P. cruentum* extracts by HPLC-DAD-APCI-QTOF-MS/MS analysis, including peak annotation, high-resolution mass spectrometry features and UV–Vis maxima.

Peak	RT (min)	Identification	Experimental $[M + H]^+$ *m/z*	UV–Vis maxima (nm)	MS/MS product ions
1	3.471	Chlorophyll derivative I	549.4875	340s, 380s, 428	307.1400, 97.1048
2	4.177	Chlorophyll derivative II	549.4871	340s, 380s, 428	305.1354, 255.0720
3	6.658	Phaeophorbid B, methyl ester	623.2851	420	565.2795, 499.2056
4	7.347	Pheophytin derivative	909.5374	340s, 380s, 428	631.2374, 559.2071
5	7.668	Zeaxanthin isomer I	569.4357	422, 446, 474	464.8067, 281.2215, 175.1480
6	8.057	Zeaxanthin	569.4342	420s, 445, 476	423.3309, 338.2549, 175.1480
7	9.826	Zeaxanthin isomer II	569.4365	422, 446, 474	539.4249, 175.1480
8	11.920	3-Acetylpheophytin a	887.5709	410	609.2687, 549.2495
9	13.621	Divinyl pheophytin a	869.5547	410	592.2652, 487.1950
10	14.656	Plastoquinone	749.6235	420	551.3212, 495.2654, 151.0743
11	14.862	Pheophytin b	885.5527	420	607.2536, 503.2403
12	16.500	Pheophytin a	871.5739	420	594.2828, 533.2567
13	18.701	β-Carotene	537.4441	420s, 450, 480	375.2935, 173.1293
14	18.892	β-Carotene isomer	537.4456	420s, 450, 480	261.1637, 146.1004
